# Elevation of cAMP Levels Inhibits Doxorubicin-Induced Apoptosis in Pre- B ALL NALM- 6 Cells Through Induction of BAD Phosphorylation and Inhibition of P53 Accumulation

**Published:** 2015

**Authors:** Ahmad Fatemi, Ahmad Kazemi, Meysam Kashiri, Majid Safa

**Affiliations:** 1 *Department of Hematology, School of Allied Medicine, Iran University of Medical Sciences, Tehran, Iran.*; 2 *Razi Drug Research Center, Iran University of Medical Sciences, Tehran, Iran.*

**Keywords:** Doxorubicin, apoptosis, cAMP, p53, BAD

## Abstract

Recognition of the molecular mechanisms of cAMP action against DNA damage-induced apoptosis can be useful to improve the efficacy of DNA damaging therapeutic agents. Considering the critical role of bcl-2-associated death promoter (BAD) and p53 proteins in DNA damage -induced apoptosis, the aim of this study was to assess the effect of cAMP-elevating agents on these proteins in doxorubicin-treated pre-B acute lymphoblastic leukemia (pre-B ALL) NALM-6 cells.The pre-B ALL cell line NALM-6 was cultured and treated with doxorubicin in combination with or without cAMP-elevating agents forskolin and 3-isobutyl-1-methylxanthine (IBMX). Cell viability was measured by trypan blue staining and MTT assay. For evaluation of apoptosis, annexin-V staining by flow cytometry and caspase-3 activity assay were used. Protein expression of p53, BAD and phoshorylated BAD was detected by western blotting analysis.cAMP-increasing agents diminished the doxorubicin-mediated cytotoxicity in NALM-6 cells as indicated by the viability assays. Annexin-V apoptosis assay showed that the cAMP-elevating agents decreased doxorubicin-induced apoptosis. Moreover, doxorubicin-induced caspase-3 activity was attenuated in the presence of cAMP-increasing agents. Western blot results revealed the reduced expression of p53 protein in cells treated with combination of cAMP-elevating agents and doxorubicin in contrast to cells treated with doxorubicin alone. Expression of total BAD protein was not affected by doxorubicin and cAMP-elevating agents. However, phosphorylation of BAD protein was induced in the presence of cAMP-elevating agents. Our study suggests that elevated cAMP levels inhibit doxorubicin-induced apoptosis in pre-B ALL cells through induction of BAD phosphorylation and abrogation of p53 accumulation.

Chemotherapeutic DNA-damaging agents such as doxorubicin are commonly used in the treatment of a wide range of cancers, including hematological malignancies. The primary mecha-nism of action of these agents is to induce apoptosis (1, 2). Apoptosis is a programmed cell death process which destroys cells that are damaged irreparably by internal (e.g. DNA damage) or external (e.g. an extracellular death ligand) stimuli. The regulation of apoptosis is crucial for controlling development and tissue homeostasis, defending against pathogens and preventing tumor formation and proliferation. Several gene products have been identified to be critical in the regulation of apoptosis including p53 and Bcl-2 family proteins (3-5). The p53 tumor suppressor protein is an essential component of the DNA damage response pathway in the normal cells. Under normal conditions, p53 protein is maintained at low levels in the cell via proteasome-mediated degradation. Under different stress conditions such as DNA damage, p53 is stabilized and accumulates in the nucleus where it transactivates genes mediating cell cycle arrest, senescence, apoptosis and DNA repair. Cell type, and the nature and context of DNA damage influence the outcome of p53 activation (6, 7). Bcl-2 family proteins are key regulators of apoptosis. On the basis of functional features, this family is divided into two subfamilies: anti-apoptotic (e.g. Bcl-2, Bcl-XL) and pro- apoptotic (e.g. Bax, Bak, Bok, Bad) proteins. The Bcl-2-associated death promoter (BAD) is a pro-apoptotic member of this family which is involved in promotion of apoptosis (in dephosphorylated form) through binding and inhibition of Bcl-2 and Bcl-XL anti-apoptotic proteins. Phosphorylated BAD (Ser 75, 99 and 118) interacts with 14-3-3 proteins instead of Bcl-2 or Bcl-XL, resulting in inactivation of BAD and inhibition of apoptosis (4, 8, 9). Since expression and phosphorylation of p53 and BAD are key processes in apoptosis regulation, it is of great importance to dissect the mechanisms and factors that could influence these processes. Cyclic adenosine monophosphate (cAMP) is an important second messenger that its generation is induced by stimulation of G protein-coupled receptors and activation of adenylyl cyclase. The cAMP signaling pathway regulates a multitude of cellular functions, including proliferation, differentiation and apoptosis (10, 11). Depending on the cell type and nature of death- inducing signal, diverging effects of cAMP on cell survival have been demonstrated (12-15). In many biological systems, the induction of apoptosis requires elevated cAMP. Exposure to cAMP induces apoptosis in ovarian granulosa cells and B-cell chronic lymphocytic leukemia cells (16, 17). However, elevated cAMP also inhibits apoptosis in a variety of other systems, such as promonocytic leukemia cells exposed to chemotherapeutic drugs (18) and macrophages exposed to nitric oxide (19). These data indicate cell-type specificity underlying cAMP signaling.

In this study, the effect of cAMP- elevating agents on expression of p53 and BAD proteins as well as phoshorylated BAD protein was investigated in doxorubicin-treated pre-B ALL NALM-6 cell line. We demonstrated that an elevated cAMP level inhibits doxorubicin-induced apoptosis in NALM-6 cells through phospho-rylation of BAD protein and down- regulation of p53.

## Materials and methods

***Cell culture and drug treatment***

Pre- B ALL cell line NALM-6 was cultured in suspension in RPMI 1640 medium supplemented with 2 mM L- glutamine, 10% fetal bovine serum (FBS), 100 units/ ml penicillin, and 100 μg/ ml streptomycin in a humidified atmosphere of 5% CO2 at 37 °C. For drug treatment, cells were seeded into a 6- well culture plate at a density of 5×10^5^ cells/ well and treated with cAMP-increasing agents (50 μM forskolin and 100 μM 3-isobutyl-1-methylxanthine (IBMX)). After 30 min, the pretreated cells were incubated with doxorubicin (250 nM and 500 nM) for 24 h.

***Cell viability measurement by trypan blue exclusion and MTT assays***

To investigate the effect of doxorubicin in the presence or absence of cAMP- increasing agents (forskolin and IBMX) on cell viability, NALM-6 cells were subjected to trypan blue exclusion assay and MTT colorimetric method. For trypan blue staining, 24 h after treatment the cell suspension was mixed with 0.4% trypan blue solution at a 1:1 ratio. After 1-2 min incubation at room temperature, the mixture was loaded onto one chamber of Neubauer hemocytometer and squares of the chamber were observed under a light microscope. The viable/ live (clear) and non-viable/ dead (blue) cells were counted and the viability was calculated using the formula (number of live cells counted/ total number of cells counted)×100. For MTT assay, NALM-6 cells were seeded into a 96- well culture plate (5000 cells/ well) and treated with doxorubicin and cAMP-elevating agents. 24 h after treatment, the medium was removed and cells were incubated with MTT solution (5 mg/ ml in PBS) for 4 h at 37 °C. The resulting formazan cristals were solubilized by addition of 100 μl dimethylsulfoxide (DMSO) at each well, and the absorbance was measured at 570 nm by ELISA reader.

**Phosphatidylserine (PS) externalization (annexin -V assay)**

The effect of cAMP- increasing agents on doxorubicin- induced cell death was assayed by apoptosis analysis. In brief, 24 h after treatment NALM-6 cells were collected and washed with PBS, and 10^6^ cells per sample were resuspended in 100 μl incubation buffer. After addition of annexin V-FITC reagent (2 μl per sample), the cell suspension was incubated for 10 min in the dark at room temperature, and fluorescence was measured using Becton–Dickinson FACS. AnnexinV- positive cells were considered to be in apoptotic phase.

***Caspase- 3 activity assay***

To evaluate caspase -3 activity, cell lysates were prepared after their respective treatment with doxorubicin in combination with or without forskolin and IBMX. Briefly, the cells were treated with the indicated agents, and the cell lysates were prepared. The reaction mixture (total volume, 100 µl) contained 20 µg of cell lysate and 10 µl of the caspase -3 substrate acetyl-Asp-Glu-Val-Asp-p-nitroanilide (Ac-DEVD-pNA) was incubated in a 96- well plate at 37 °C for 2 h. The absorbances of samples were then read at 405 nm in an ELISA reader.

***Western blot analysis***

Four hour After treatment, the cells were collected and lysed (5 × 10^6^ cells/ aliquots) in RIPA buffer (10 mM Tris–HCl, pH 7.4, 150 mM NaCl, 5 mM EDTA, 1% Triton X-100, 0.1% sodium dodecyl sulfate, and 0.5% sodium deoxycholate) containing protease and phosphatase inhibitor cocktails (Sigma ). The cell lysate was centrifuged at 13000 g for 20 min at 4 °C and the supernatant was collected. Total protein of the supernatant was quantified using the Bradford protein assay and equal amounts of total cellular protein were run on a 10% SDS–PAGE. The resolved proteins were then transferred from the gel to a nitrocellulose membrane (Hybond-ECL, Amersham Corp).  Afterward, the membrane was incubated in blocking buffer (1xTris buffered saline [TBS], 0.1% Tween- 20 with 5% nonfat dry milk) for 1 h, and probed overnight at 4 °C with specific primary antibodies (Cell Signaling Technology, UK) against p53 (2527), Bad (9239), phospho-Bad (Ser118) (9297), phospho-Bad (Ser99) (5286), phospho-Bad (Ser75) (5284). After washing five times in 1xTBS containing 0.1% Tween-20 (TBST), the membrane was incubated for 1 hat room temperature with horse radish peroxidase (HRP)-conjugated secondary antibody (anti rabbit antibody) (7074P2). The immunoreactive proteins were then visualized with a chemiluminescence detection system (Amersham ECL Advance Kit, GE Healthcare ) according to the manufacturer’s protocol.

***Statistical analysis***

Statistical analysis was done using SPSS 12. Twotailed Student’s t test was used to determine if there is a significant difference between experimental variables. A P- value <0.05 was considered as statistically significant.

## Results

**Elevated level of cAMP diminished the doxorubicin- induced apoptosis in NALM-6 cells**

**Fig. 1:**
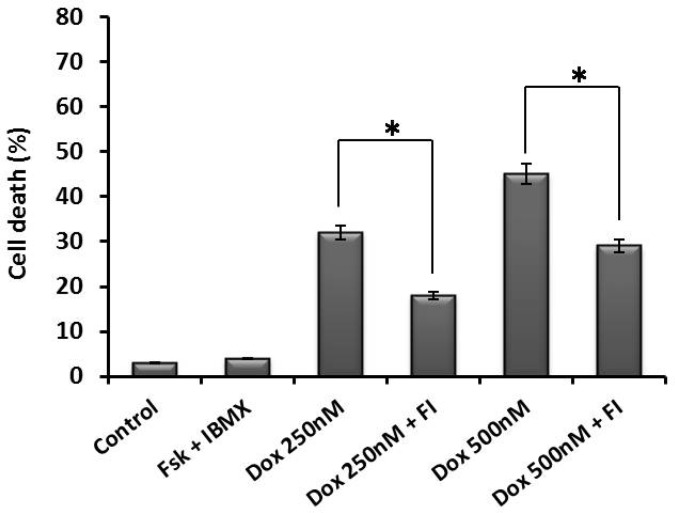
Doxorubicin-induced cell death was attenuated by cAMP increasing agents. NALM-6 cells were treated with forskolin and IBMX (FI). After 30 min, the pretreated cells were incubated with doxorubicin (250 nM and 500 nM) for 24 h and then cell viability was assessed using trypan blue exclusion assay. Elevated cAMP significantly decreased cell death (* P< 0.05).

**Fig. 2:**
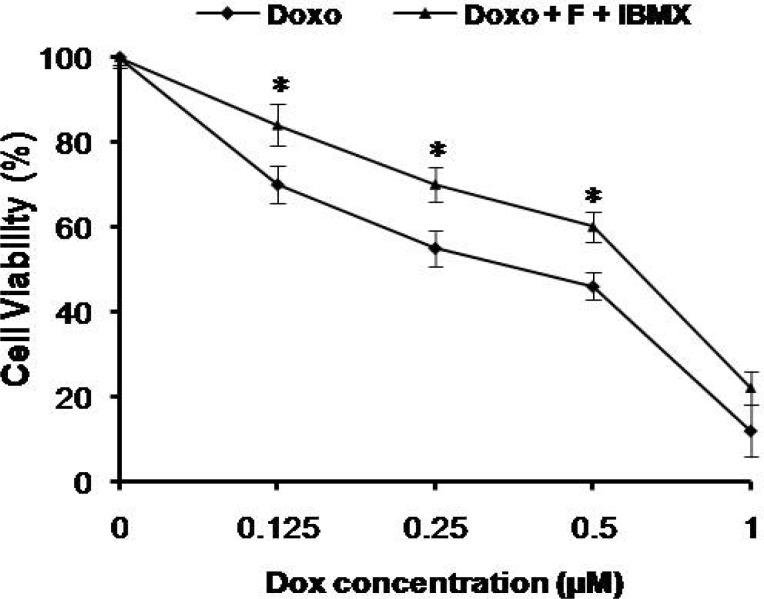
cAMP-increasing agents attenuated the cytotoxic effects of doxorubicin in NALM-6 cells. NALM-6 cells were pretreated with forskolin and IBMX (FI) 30 min prior to the addition of doxorubicin and cell viability was assessed using MTT assay 24 h after treatment. (mean± SEM, n = 3). Elevated cAMP significantly decreased cell death (* P <0.05; relative to cells treated with doxorubicin only

**Fig. 3:**
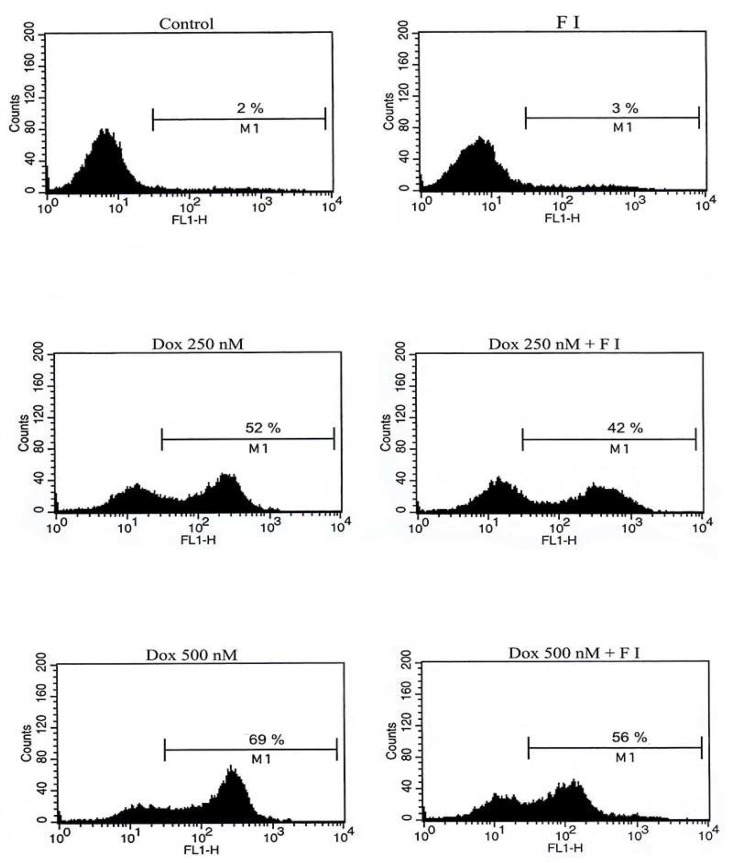
Doxorubicin-induced apoptosis was diminished by cAMP increasing agents. NALM-6 cells were treated with forskolin and IBMX (FI). After 30 min, the pretreated cells were incubated with doxorubicin (250 nM and 500 nM) for 24 h. The cells were then analyzed for annexin-V uptake by FACS

**Fig 4:**
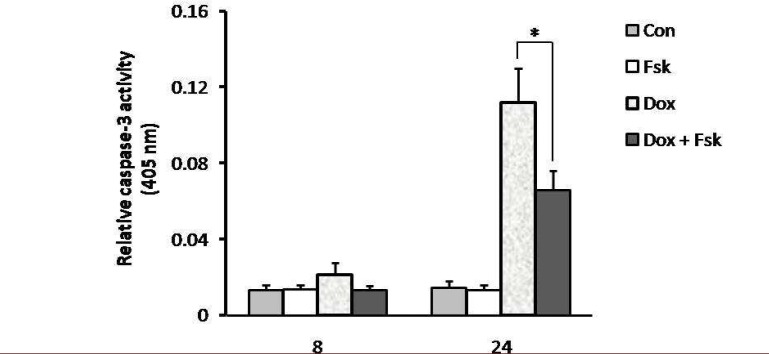
Doxorubicin-induced caspase activation was diminished by cAMP-increasing agents. NALM-6 cells were treated with forskolin and IBMX (FI). After 30 min, the cells were incubated with doxorubicin (250 nM) for 8 and 24 h. The cells were then assayed for caspase-3 protease activity (* P<0.05

Cytotoxicity of doxorubicin in the presence or absence of cAMP-increasing agents (forskolin and IBMX) was determined by trypan blue exclusion and MTT assay. Forskolin activates adenylyl cyclase, and IBMX is an inhibitor of the phosphodiesterase which converts cAMP to AMP, resulting in elevated cAMP levels within the cell (20). Viability of NALM-6 cells after exposure to doxorubicin was reduced in a dose dependent manner with 50% reduction in viability at 500 nM concentration of the drug. As shown in Fig. 1 (trypan blue exclusion assay) and Fig. 2 (MTT assay), cAMP-increasing agents attenuated the doxorubicin-mediated cytotoxicity in NALM-6 cells. To investigate the effect of elevated level of cAMP on doxorubicin-induced apoptosis, NALM-6 cells were assayed for annexin V staining and caspase-3 protease activity. As shown in Fig. 3, pretreatment of cells with cAMP-increasing agents was associated with considerable lower percentages of annexin-V positive cells (apoptotic cells) in comparison to the cells treated with doxorubicin only. Moreover, cotreatment of cells with doxorubicin and cAMP-increasing agents resulted in significant attenuation of the doxorubicin-induced caspase-3 activation (Fig. 4). These findings indicate the inhibitory effect of cAMP levels on doxorubicin- induced apoptosis in NALM- 6 cells.

**Elevated levels of cAMP diminished doxorubicin -induced accumulation of p53 protein in NALM-6 cells**

**Fig 5:**
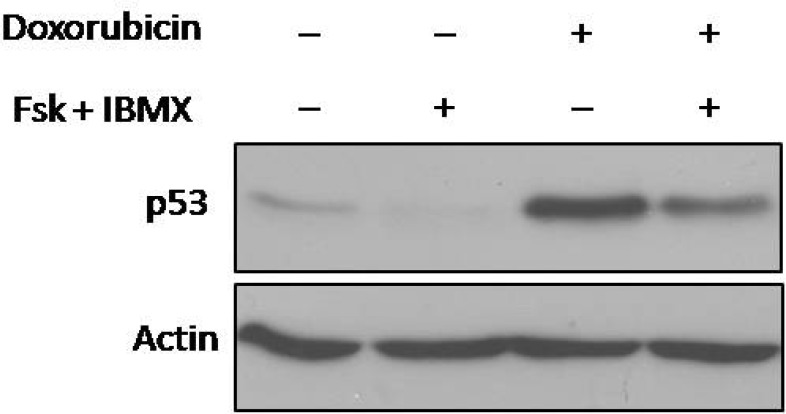
Effect of cAMP levels on total p53 protein expression upon DNA damage. NALM-6 cells were treated with forskolin and IBMX (FI). After 30 min, the pretreated cells were incubated with doxorubicin for 4 h and then protein expression was assessed using western blot analysis. Elevated cAMP decreased p53 expression induced by doxorubicin in NALM-6 cells

NALM-6 cells accumulate p53 protein after DNA damage induced by doxorubicin. To evaluate whether doxorubicin-induced accumulation of p53 is affected by cAMP signaling pathway, protein expression of p53 was assessed after exposure of NALM-6 cells with doxorubicin in the presence or absence of forskolin / IBMX. As shown in Fig. 5, elevated levels of cAMP reduced p53 accumulation. 

**Fig. 6:**
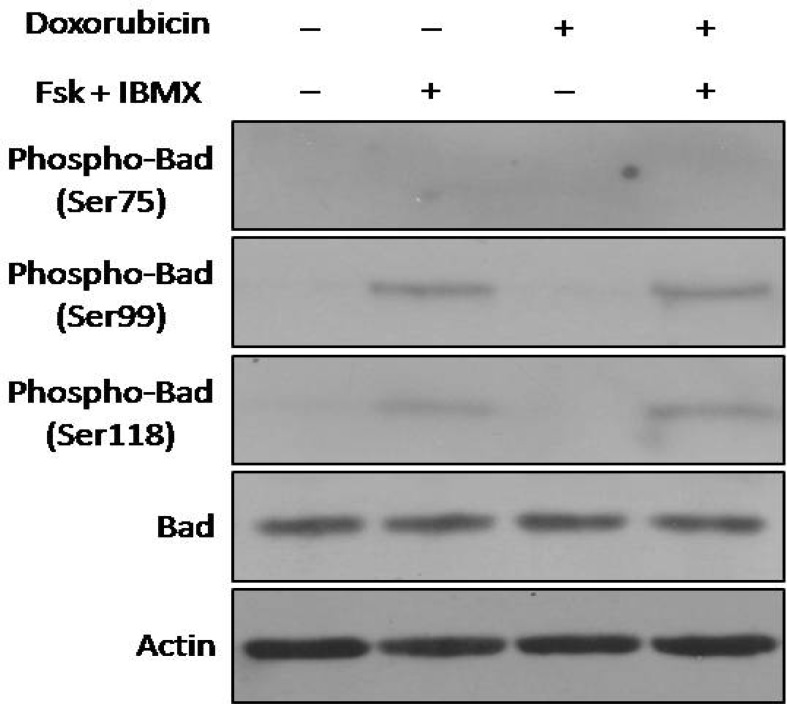
Effect of cAMP levels on BAD expression and phosphorylation. NALM-6 cells were treated with forskolin and IBMX. After 30 min, the pretreated cells were incubated with doxorubicin for 4 h and then protein expression was assessed using western blot analysis. Elevated cAMP increased BAD phosphorylation at ser 99 and ser 118, but no ser 75. There was no difference in expression of total BAD proteins

**cAMP increasing agents increased phosphor-ylation state of BAD at serines 99 and 118 in doxorubicin-treated NALM- 6 cells**

To investigate the effect of cAMP increasing agents on phosphorylation of BAD after doxorubicin- induced DNA damage, three serine residues (Ser75, 99 and 118) were evaluated after treatment of NALM-6 cells with doxorubicin in the presence or absence of cAMP-increasing agents. Significantly, increased phosphorylation of BAD on 99 and 118 serine sites were seen in the cells pretreated with forskolin/IBMX (see fig. 6). Expression of total BAD proteins and phosphorylation of BAD on Ser75 were not affected by doxorubicin and cAMP- elevating agents.

## Discussion

In this study, we showed the inhibitory effect of cAMP on DNA damage-induced apoptosis in pre B ALL cell line NALM-6. In addition, we found that cAMP exerts this inhibitory effect through abrogation of p53 accumulation as well as induction of BAD phosphorylation.

The capacity of cAMP- increasing agents to regulate apoptosis has been examined in different cell types, in many cases with conflicting results. While a number of studies have reported the inhibitory effect of cAMP on apoptosis in different cell types (12, 18, 19, 21), other studies indicated that cAMP signaling pathway causes apoptosis or potentiates its induction by other agents (13, 22, 23). Depending on the cell type and nature of death-inducing signal, diverging effects of cAMP on cell survival have been demonstrated. In this study, we showed the inhibitory effect of cAMP on doxorubicin-induced apoptosis in pre B ALL cell line NALM-6 (Figures 3 and 4). This finding is consistent with previous studies that cAMP decreased chemotherapeutics- induced apoptosis in cancer cells (18, 24).

Regarding the inhibitory effect of cAMP against chemotherapeutic drugs- induced apoptosis, identification of the molecular mecha sm(s) underlying cAMP- mediated modulation of apoptosis is of particular importance.

P53 is a tumor suppressor known as major factor in DNA damage-induced apoptosis and loss of p53 that commonly occurs in tumors is thought to be a way to escape from apoptosis. Abrogation of p53 pathway can be caused by genetic mutation (mutated *P53*) or indirect mechanisms that suppress wild-type p53 (6, 7). Because the majority of hematologic malignancies express the wild-type p53 (25, 26), it is of great importance to identify the indirect mechanisms to improve killing of these tumor cells by DNA-damaging therapeutic agents. Previous studies have been demonstrated that cAMP attenuates p53 protein levels through inhibition of posttranslational modifications (24, 27). Consistent with previous studies, our finding showed that increased levels of cAMP abrogate total p53 accumulation after exposure of NALM-6 cells to doxorubicin (Fig. 5). This finding can introduce the aberrant activation of cAMP signaling pathway as an indirect mechanism that is involved in wild-type p53 suppression.

Nuclear factor-κB (NF-κB) consists of a family of transcription factors that play critical roles in inflammation, cell proliferation, differentiation, and survival. In response to various stimuli, such as DNA damage, NF-κB is activated, binds to DNA and regulates the expression of a variety of genes, including antiapoptotic genes (28). The constitutive and deregulated activation of NF-κB found in many solid tumors as well as hematological malignancies is believed to promote cell survival and confer treatment resistance (29-31). Previous studies have shown that cAMP potentiates the DNA damage-induced NF-κB activation (32, 33). Based on these reports, cAMP attenuates the DNA damage-induced apoptosis through NF-κB activation.

Interestingly, we found a new mechanism for cAMP-mediated inhibition of doxorubicin-induced apoptosis that involves BAD protein. BAD is a pro-apoptotic member of Bcl-2 family which is regulated by posttranslational modifications. BAD binds to the anti-apoptotic proteins Bcl-2 and Bcl-XL and neutralizes the anti-apoptotic effects of these proteins. Phosphorylation of BAD induces its dissociation from Bcl-2/ Bcl-XL, resulting in liberation of these anti-apoptotic proteins, which can then interact with Bax to inhibit apoptosis. Phosphorylation sites of the human proapoptotic protein BAD are Ser 75, Ser 99 and Ser 118 (4, 8, 9). Our results showed that cAMP induces phosphorylation of BAD at Serines 99 and 118 that can decrease doxorubicin-induced apoptosis in NALM-6 cells (Fig. 6). However, total expression of BAD protein was not affected in the presence of cAMP-increasing agents. Previous reports have shown different sites of BAD phosphorylation induced by cAMP pathway. Consistent with our result, Lizcano et al. showed that cAMP-dependent regulation of BAD is mediated via phosphorylation at Ser 118 in HEK-293 cells. They found that Ser 118 of protein BAD is phosphorylated preferentially by protein kinase A (PKA) in cells exposed to cAMP-elevating agents (4). Moreover, studies on myeloid leukemia cells exposed to cAMP-elevating agents also demonstrated PKA-induced phosphorylation of BAD at Ser118 site (34, 35).In conclusion, the present study indicated that elevated cAMP levels may abrogate doxorubicin-induced apoptosis in pre-B ALL cells through a new mechanism involving the induction of BAD phosphorylation. Our results also demonstrated that cAMP-increasing agents inhibit p53 accumulation during the DNA damage induced by doxorubicin. On the basis of these findings, cAMP can be considered as a survival factor in pre-B ALL cells exposed to doxorubicin. Therefore, inhibitors of cAMP signaling pathway might prove beneficial in treatment of pre-B ALL tumors, at least for patients with wild-type p53 cells undergoing curative antineoplastic therapy with DNA- damaging agents such as doxorubicin.

## References

[1] Martin A, Morgan E, Hijiya N (2012). Relapsed or refractory pediatric acute lymphoblastic leukemia: current and emerging treatments. Paediatr Drugs.

[2] Stanulla M, Schrappe M (2009). Treatment of childhood acute lymphoblastic leukemia. Semin Hematol.

[3] Arends MJ, Wyllie AH (1991). Apoptosis: mechanisms and roles in pathology. Int Rev Exp Pathol.

[4] Lizcano JM, Morrice N, Cohen P (2000). Regulation of BAD by cAMP-dependent protein kinase is mediated via phosphorylation of a novel site, Ser155. Biochem J.

[5] Rai NK, Tripathi K, Sharma D (2005). Apoptosis: a basic physiologic process in wound healing. Int J Low Extrem Wounds.

[6] Vogelstein B, Lane D, Levine AJ (2000). Surfing the p53 network. Nature.

[7] Vousden KH, Lane DP (2007). p53 in health and disease. Nat Rev Mol Cell Biol.

[8] Yang E, Zha J, Jockel J (1995). Bad, a heterodimeric partner for Bcl-XL and Bcl-2, displaces Bax and promotes cell death. Cell.

[9] Zha J, Harada H, Yang E (1996). Serine phosphorylation of death agonist BAD in response to survival factor results in binding to 14-3-3 not BCL-X(L). Cell.

[10] Cheng X, Ji Z, Tsalkova T (2008). Epac and PKA: a tale of two intracellular cAMP receptors. Acta Biochim Biophys Sin (Shanghai).

[11] Fimia GM, Sassone-Corsi P (2001). Cyclic AMP signalling. J Cell Sci.

[12] Grandoch M, Bujok V, Fleckenstein D (2009). Epac inhibits apoptosis of human leukocytes. J Leukoc Biol.

[13] Lomo J, Blomhoff HK, Beiske K (1995). TGF-beta 1 and cyclic AMP promote apoptosis in resting human B lymphocytes. J Immunol.

[14] Rodriguez G, Ross JA, Nagy ZS (2013). Forskolin-inducible cAMP pathway negatively regulates T-cell proliferation by uncoupling the interleukin-2 receptor complex. J Biol Chem.

[15] Mitra A, Ross JA, Rodriguez G (2012). Signal transducer and activator of transcription 5b (Stat5b) serine 193 is a novel cytokine-induced phospho-regulatory site that is constitutively activated in primary hematopoietic malignancies. J Biol Chem.

[16] Tiwari S, Felekkis K, Moon EY (2004). Among circulating hematopoietic cells, B-CLL uniquely expresses functional EPAC1, but EPAC1-mediated Rap1 activation does not account for PDE4 inhibitor-induced apoptosis. Blood.

[17] Hosokawa K, Aharoni D, Dantes A (1998). Modulation of Mdm2 expression and p53-induced apoptosis in immortalized human ovarian granulosa cells. Endocrinology.

[18] Garcia-Bermejo L, Perez C, Vilaboa NE (1998). cAMP increasing agents attenuate the generation of apoptosis by etoposide in promonocytic leukemia cells. J Cell Sci.

[19] von Knethen A, Brune B (2000). Attenuation of macrophage apoptosis by the cAMP-signaling system. Mol Cell Biochem.

[20] Insel PA, Ostrom RS (2003). Forskolin as a tool for examining adenylyl cyclase expression, regulation, and G protein signaling. Cell Mol Neurobiol.

[21] Lee MR, Liou ML, Yang YF (1993). cAMP analogs prevent activation-induced apoptosis of T cell hybridomas. J Immunol.

[22] McConkey DJ, Orrenius S, Okret S (1993). Cyclic AMP potentiates glucocorticoid-induced endogenous endonuclease activation in thymocytes. FASEB J.

[23] Vucic V, Niciforovic A, Adzic M (2008). The combination of gamma ionizing radiation and 8-Cl-cAMP induces synergistic cell growth inhibition and induction of apoptosis in human prostate cancer cells. Invest New Drugs.

[24] Safa M, Kazemi A, Zaker F (2011). Cyclic AMP-induced p53 destabilization is independent of EPAC in pre-B acute lymphoblastic leukemia cells in vitro. J Recept Signal Transduct Res.

[25] Secchiero P, di Iasio MG, Gonelli A (2008). The MDM2 inhibitor Nutlins as an innovative therapeutic tool for the treatment of haematological malignancies. Curr Pharm Des.

[26] Stuhmer T, Bargou RC (2006). Selective pharmacologic activation of the p53-dependent pathway as a therapeutic strategy for hematologic malignancies. Cell Cycle.

[27] Safa M, Kazemi A, Zand H (2010). Inhibitory role of cAMP on doxorubicin-induced apoptosis in pre-B ALL cells through dephosphorylation of p53 serine residues. Apoptosis.

[28] Janssens S, Tschopp J (2006). Signals from within: the DNA-damage-induced NF-kappaB response. Cell Death Differ.

[29] Basseres DS, Baldwin AS (2006). Nuclear factor-kappaB and inhibitor of kappaB kinase pathways in oncogenic initiation and progression. Oncogene.

[30] Nakanishi C, Toi M (2005). Nuclear factor-kappaB inhibitors as sensitizers to anticancer drugs. Nat Rev Cancer.

[31] Packham G (2008). The role of NF-kappaB in lymphoid malignancies. Br J Haematol.

[32] Safa M, Zand H, Mousavizadeh K (2010). Elevation of cyclic AMP causes an imbalance between NF-kappaB and p53 in NALM-6 cells treated by doxorubicin. FEBS Lett.

[33] Kloster MM, Naderi EH, Carlsen H (2011). Hyperactivation of NF-kappaB via the MEK signaling is indispensable for the inhibitory effect of cAMP on DNA damage-induced cell death. Mol Cancer.

[34] Gausdal G, Wergeland A, Skavland J (2013). Cyclic AMP can promote APL progression and protect myeloid leukemia cells against anthracycline-induced apoptosis. Cell Death Dis.

[35] Safa M, Mousavizadeh K, Noori S (2014). cAMP protects acute promyelocytic leukemia cells from arsenic trioxide-induced caspase-3 activation and apoptosis. Eur J Pharmacol.

